# Phenotype versus genotype discordant rifampicin susceptibility testing in tuberculosis: implications for a diagnostic accuracy

**DOI:** 10.1128/spectrum.01631-23

**Published:** 2023-11-20

**Authors:** Mehmood Qadir, Rani Faryal, Muhammad Tahir Khan, Sajjad Ahmed Khan, Shulin Zhang, Weimin Li, Dong Qing Wei, Sabira Tahseen, Timothy D. McHugh

**Affiliations:** 1 National TB Control Program, National TB Reference Laboratory, Islamabad, Pakistan; 2 Department of Microbiology, Quaid-i-Azam University, Islamabad, Pakistan; 3 Zhongjing Research and Industrialization Institute of Chinese Medicine, Zhongguancun Scientific Park, Nanyang, Henan, China; 4 Institute of Molecular Biology and Biotechnology (IMBB), The University of Lahore, Lahore, Pakistan; 5 School of Medicine, Department of Immunology and Microbiology, Shanghai Jiao Tong University, Shanghai, China; 6 National Tuberculosis Clinical Lab of China, Beijing Chest Hospital, Capital Medical University, Beijing, China; 7 School of Life Sciences and Biotechnology, Shanghai Jiao Tong University, Shanghai, China; 8 Peng Cheng Laboratory, Shenzhen, Guangdong, China; 9 Centre for Clinical Microbiology, University College London, London, United Kingdom; Weill Cornell Medicine, New York, New York, USA

**Keywords:** *M. tuberculosis*, DST, rifampicin, resistance, discordance, *rpoB*

## Abstract

The World Health Organization recommends culture-based techniques as the gold standard for rifampicin-resistant (RR) tuberculosis. However, as rapid molecular assays are rolled out, discordance in resistance has been observed. The current study compared 516 clinical isolates on Lowenstein–Jenson (LJ) and Mycobacteria Growth Indicator Tube (MGIT) drug susceptibility testings (DSTs) with *rpoB* sequencing. The discordance rate was higher in MGIT DST (24%) compared to LJ (9.4%). We report discordant results for RpoB mutations 430P (MGIT: 14/20; LJ: 19/20), 435Y (MGIT: 5/21; LJ: 17/21), 445N (MGIT: 11/18; LJ: 15/18), 445L (MGIT: 0/9; LJ: 8/9), 452P (MGIT: 5/20; LJ: 17/20), and 445C (MGIT: 1/6; LJ: 2/6) on LJ and MGIT DSTs, respectively. Mutations N438K and T444A, sensitive on LJ and MGIT DSTs, are novel. Two novel discordant mutations, Q436H+N437del and S428I, were also detected. One novel large deletion (Del) ggaccagaacaacccg/G (Del of DQNNP at positions 435–439) demonstrated concordance on LJ and MGIT DST. Numerous double and triple mutations including some novel combinations, M434V+H445N, L430R+D435Y, H445N+S450W, S428R+L430P, M434L+H445N, D435G+A451V, and M434I+D435G+A451V, have also shown higher discordant results on MGIT DST. The discordance results of RR are higher in MGIT DST compared to LJ. We suggest that the genotypic result should be preferred instead of phenotypic DST in case of discordant mutations. Physicians should be aware of such discordance to prescribe an effective treatment. Untargeted whole-genome sequencing to capture MGIT-sensitive and large deletions may be reconsidered for better treatment outcomes in high-burden countries.

**IMPORTANCE:**

An accurate diagnosis of drug resistance in clinical isolates is an important step for better treatment outcomes. The current study observed a higher discordance rate of rifampicin resistance on Mycobacteria Growth Indicator Tube (MGIT) drug susceptibility testing (DST) than Lowenstein–Jenson (LJ) DST when compared with the *rpoB* sequencing. We detected a few novel mutations and their combination in rifampicin resistance isolates that were missed by MGIT DST and may be useful for the better management of tuberculosis (TB) treatment outcomes. Few novel deletions in clinical isolates necessitate the importance of *rpoB* sequencing in large data sets in geographic-specific locations, especially high-burden countries. We explored the discordance rate on MGIT and LJ, which is important for the clinical management of rifampicin resistance to avoid the mistreatment of drug-resistant TB. Furthermore, MGIT-sensitive isolates may be subjected to molecular methods of diagnosis for further confirmation and treatment options.

## INTRODUCTION

Tuberculosis (TB) is among the top 10 most common causes of death from a single infectious agent called *Mycobacterium tuberculosis* (*M. tuberculosis*). The resistance to two first-line drugs against *M. tuberculosis*, constituting a standard therapy, isoniazid (INH) and rifampicin (RIF), is rising. The multidrug-resistant (MDR)-TB (defined as resistance to both INH and RIF) (1, 2) has further complicated the TB therapy by controlling about half a million new MDR-TB per year. An estimated 3.4% of new and 18% of previously treated cases were rifampicin-resistant (RR) or MDR-TB globally (3). According to the latest TB report, MDR-TB or RR-TB (MDR/RR-TB) cases were relatively stable from 2015 to 2020 but increased in 2021 (*n* = 450,000), showing a 3.1% rise from 2020 (*n* = 437,000) (4). In Pakistan, an estimated 4.2% of new TB cases were MDR/RR-TB in 16% of previously treated patients (5).

Culture-based methods, rapid molecular tests, and whole-genome sequencing (WGS) technologies are required for biological confirmation of MDR/RR-TB (6 – 8). Phenotypic drug susceptibility testings (DSTs) by Lowenstein–Jenson (LJ) and liquid medium-based Mycobacteria Growth Indicator Tube (MGIT) (BD Bactec MGIT, Becton, Dickinson, USA) are the World Health Organization (WHO)-endorsed phenotypic methods (9). However, technological advances have developed various molecular-based assays to detect targeted resistance-conferring mutations to different anti-TB drugs. GeneXpert MTB/RIF (Cepheid Inc., Sunnyvale, CA, USA) and MTBDRplus (Hain Lifescience, Nehren, Germany) line probe assays are the most commonly used to rapidly detect rifampicin resistance in *M. tuberculosis* (10 – 12). These molecular methods have been developed to detect a target region in the *rpoB* gene, known as 81 bp hot-spot region (codons 426–452), also called rifampicin resistance-determining region (RRDR). Mutations in RRDR of *rpoB* help to rapidly identify resistance and inform good clinical decisions (10, 12 – 14). However, less prevalent and geographic-specific mutations not covered by the endorsed molecular assays and other unknown mechanisms of resistance to rifampicin, including compensatory mutations, necessitate the phenotypic methods to confirm resistance. Previous studies have shown that low-level rifampicin resistance, which has clinical relevance, may be missed by the standard phenotypic method MGIT DST (9, 15 – 19). The result is often recorded in discordance between phenotypic and genotypic methods. Such mutations are discordant (9, 15 – 19).

MGIT DST is routinely performed on a WHO-defined critical concentration of 1 µg/mL in defined conditions. However, at a critical concentration of 1 µg/mL, it has been shown that strains harboring discordant mutations are detected as rifampicin-susceptible as this critical concentration is above the epidemiological cutoff (ECOFF) values (20). Discordant mutations and the consequent discordance between LJ DST, MGIT DST, and genotypic DST may lead to uncertainty in the appropriate TB management and make it hard to treat the patients. The diagnostic accuracy of phenotypic and genotypic assays varies as the frequency of mutations in different geographic regions differs (21, 22).

Recently, the WHO has revised the rifampicin critical concentration from 1 to 0.5 µg/mL (23) on MGIT DST to reduce this issue; however, this study was conducted before the revised rifampicin critical concentration.

Pakistan has been ranked fifth among 30 high-burden countries; data on discordant are not available for better TB management. Phenotypic DST and MTBDRplus are performed on all cases initially diagnosed as RR by GeneXpert to identify other susceptible drugs that can be included in the regimen and for the confirmation of GeneXpert results. Discordant results have been encountered in our region, causing clinical and diagnostic dilemmas. For the first time, the current study was designed to identify the extent of discordant RR cases on LJ and MGIT DSTs and design strategies for accurately detecting resistance, leading to better treatment options.

## MATERIALS AND METHODS

### Sampling

All clinical samples from January 2014 to October 2016 included all available discordant isolates RR on LJ and susceptible on MGIT DST or sensitive on MGIT and LJ or RR on LJ, and MGIT was isolated in the National Tuberculosis Reference Laboratory Pakistan followed by a random selection of concordant isolates before and after the discordant isolate for this study. The National TB Reference Laboratory receives all RR cases from Punjab, the largest province of Pakistan, Islamabad capital territory, Kashmir, and Gilgit Baltistan for comprehensive phenotypic DST results.

### Specimen processing

Clinical specimens were decontaminated using the NALC-NaOH method and inoculated on Middlebrook 7H9 broth (Bactec MGIT 960 tube) LJ media for *Mycobacterium tuberculosis* complex (MTBC) growth. A rapid BD MGIT TBc identification test was performed on positive cultures to confirm MTBC. Liquid culture-based DST was performed on isolated MTBC cultures on BD Bactec MGIT 960 using the SIRE kit (Bactec MGIT 960 SIRE Kit) (24). The standard LJ proportion method for susceptibility testing of *M. tuberculosis* isolates was also performed as per the standard procedure (25) using rifampicin at 40 µg/mL. A susceptible strain, H37Rv, and two known rifampicin-resistant strains selected from National Proficiency testing round 19 received from the Supranational Reference Laboratory, Antwerp, Belgium, were used for quality assessment and accuracy in DST results (26, 27).

### DNA extraction

DNA for polymerase chain reaction (PCR) was extracted from 516 randomly selected RR isolates including 120 *rpoB* wild type (sensitive on MGIT and LJ) and 396 RR by heating bacterial colonies at 90°C for 20 minutes in 100 µL of water followed by sonication on an ELMA E30H Sonicator (Elma Schmidbauer GmbH) for 15 minutes.

### Polymerase chain reaction

PCR was performed using 25 µL of Qiagen HotStarTaq Master Mix containing 2.5 units of HotStarTaq DNA polymerase 1× PCR buffer (contains 1.5 mM MgCl_2_) and 200 µM of each dNTP, 2 µL of each forward and reverse 10 µM primers, 8 µL of DNA, and 5 µL of water with a total volume of 50 µL in a GeneAmp PCR system 9700 (Applied Biosystems). The following cycling conditions were used: 15-minute hot start followed by 45 cycles at 94°C for 45 seconds; annealing and extension for 1 minute and 30 seconds at 72°C; and a final amplification at 72°C for 10 minutes. Primers rpoBgeneSA and rpoBgeneRB (5-GGTTCGCCGCGCTGGCGCGAAT-3) and (5-GACCTCCTCGATGACGCCGCTTTCT-3) (28) were used for the amplification of *rpoB* gene (28). PCR amplicons were analyzed on 2% agarose gel.

### Sequencing

The PCR product of *rpoB* was sequenced using the primers forward 5′-GGGAGCGGATGACCACCCA-3′ and reverse 5′- GCGGTACGGCGTTTCGATGAAC-3′ (29) (amplicon size 350 nt) on a 3130 genetic analyzer by Applied Biosystems using the BigDye Terminator v3.1 cycle sequencing kit. Sequence results were analyzed using Molecular Evolutionary Genetics Analysis version 7 (MEGA 7) (30) compared to the H37Rv RpoB gene (Rv0667) Ref-Sequence (Accession NC_000962). The confidence interval (CI) was calculated for each mutation using an online calculator (https://www.calculator.net/confidence-interval-calculator.html).

## RESULTS

Rifampicin resistance results were available on GeneXpert, LJ, and MGIT DST for 1,986 strains during the study period. A total of 1,761 were concordant, and 225 discordant isolates were analyzed (data have not been shown). The *rpoB* sequencing results were available for 516 strains, including 120 wild types and 396 (225 discordant and 171 concordance) randomly selected RR isolates (BioProject: PRJNA952823, GenBank submission ID is 2693281). The RR RpoB sequencing, LJ, and MGIT DST results are presented in Table 1; Table S1 based on the *M. tuberculosis* and *Escherichia coli rpoB* codon numbering system. We also calculated the 95% confidence interval for each mutation identified in our study to check the sensitivity of LJ and MGIT DSTs (Table 1; Table S1). Mutations were identified in 396 strains in *rpoB,* in which 42 out of 396 were susceptible in LJ DST, whereas MGIT DST reported 95 isolates as RIF-susceptible.

**TABLE 1:** Summary of rifampicin concordance and discordance on LJ and MGIT DSTs and sensitivity, stratified by RpoB mutations in *M. tuberculosis* isolates from Pakistan^*a*^

Codon change	Amino acid change *M. tuberculosis* numbering (*E. coli*)	Rifampicin DST results on LJ			Rifampicin DST results on MGIT			Total
		*R (%)*	*S* (%)	Sensitivity 95% CI	*R* (%)	*S* (%)	Sensitivity 95% CI	
**tcg→ttg**	S450L (S531L)	199 (100)	0 (0)	98.16%**–**100.00%	199 (100)	0 (0)	98.16%**–**100.00%	199
**gac→tac**	D435Y (D516Y)	16 (76.2)	5 (23.8)	52.83%**–**91.78%	4 (19)	17 (80.9)	5.45%**–**41.91%	21
**ctg→ccg**	L430P (L511P)	6 (30)	14 (70)	11.89%**–**54.28%	1 (5)	19 (95)	0.13%**–**24.87%	20
**ctg→ccg**	L452P (L533P)	15 (75)	5 (25)	50.90%**–**91.34%	3 (15)	17 (85)	3.21%**–**37.89%	20
**gac→gtc**	D435V (D516V)	19 (100)	0 (0)	82.35%**–**100.00%	19 (100)	0 (0)	82.35%**–**100.00%	19
**cac→aac**	H445N (H526N)	7 (38.9)	11 (61.1)	17.30%**–**64.25%	3 (16.6)	15 (83.3)	3.58%**–**41.42%	18
**tcg→tgg**	S450W (S531W)	15 (100)	0 (0)	78.20%**–**100.00%	15 (100)	0 (0)	78.20%**–**100.00%	15
**cac→ctc**	H445L (H526L)	9 (100)	0 (0)	66.37%**–**100.00%	1 (11.1)	8 (88.8)	0.28%**–**48.25%	9
**cac→gac**	H445D (H526D)	8 (100)	0 (0)	63.06%**–**100.00%	8 (100)	0 (0)	63.06%**–**100.00%	8
**cac→tac**	H445Y (H526Y)	8 (100)	0 (0)	63.06%**–**100.00%	8 (100)	0 (0)	63.06%**–**100.00%	8
**cac→tgc**	H445C (H526C)	5 (83.3)	1 (16.6)	35.88%**–**99.58%	4 (66.6)	2 (33.3)	22.28%**–**95.67%	6
**atc→ttc**	I491F (I572F)	5 (100)	0 (0)	47.82%**–**100.00%	4 (80)	1 (20)	28.36%**–**99.49%	5
**ins of ttc→1297**	Ins of F at 433 [F514ins (inframe)]	3 (100)	0 (0)	29.24%–100.00%	3 (100)	0 (0)	29.24%–100.00%	3
**caa→aaa**	Q432K (Q513K)	1 (100)	0 (0)	2.50%–100.00%	1 (100)	0 (0)	2.50%–100.00%	1
**caa→cca**	Q432P (Q513P)	1 (100)	0 (0)	2.50%–100.00%	1 (100)	0 (0)	2.50%–100.00%	1
**caa→cta**	Q432L (Q513L)	1 (100)	0 (0)	2.50%–100.00%	1 (100)	0 (0)	2.50%–100.00%	1
**cac→cgc**	H445R (H526R)	1 (100)	0 (0)	2.50%–100.00%	1 (100)	0 (0)	2.50%–100.00%	1
**cac→ggc**	H445G (H526G)	1 (100)	0 (0)	2.50%–100.00%	0 (0)	1 (100)	0.00%–97.50%	1
**acc→gcc**	T444A (T515A)	0 (0)	1 (100)	0.00–97.50	0 (0)	1 (100)	0.00–97.50	1
**gac→ggc**	D435G (D516G)	0 (0)	1 (100)	0.00%–97.50%	0 (0)	1 (100)	0.00%–97.50%	1
**agc→atc**	S428I (S509I)	1 (100)	0 (0)	2.50%–100.00%	0 (0)	1 (100)	0.00%–97.50%	1
**atg→ata**	M434I (M515I)	1 (100)	0 (0)	2.50%–100.00%	1 (100)	0 (0)	2.50%–100.00%	1
**gtc→ttc**	V170F (V251F)	1 (100)	0 (0)	2.50%–100.00%	1 (100)	0 (0)	2.50%–100.00%	1
**agc→ggc**	S431G (S512G)	0 (0)	1 (100)	0.00%–97.50%	0 (0)	1 (100)	0.00%–97.50%	1
**aac→aag**	N438K (N519K)	0 (0)	1 (100)	0.00%–97.50%	0 (0)	1 (100)	0.00%–97.50%	1
**ctg→cgg**	L430R (L511R)	0 (0)	2 (100)	0.00%–84.19%	0 (0)	2 (100)	0.00%–84.19%	2

^*a*^ R, resistant; S, susceptible; CI, confidence interval.

The frequency of RR isolates was higher on LJ (solid) than MGIT, as shown in Tables 1 and S1. Fully concordant results were observed between LJ and MGIT DSTs for the *M. tuberculosis* isolates harboring mutations at positions S450L (199/199), D435>V (19/19), S450>W (15/15), H445>D (8/8), H445>Y (8/8), and insertion of F at position 433 (3/3). A discordance in DST results between LJ and MGIT was detected in samples harboring mutations at positions S428I, D435Y, L430>P, L452>P, H445>N, H445>L, H445>C, H445>G, V170>F, and I491>F, respectively (Table 1; Table S1). M434V+H445N (5/5) and I491F (5/5) were all RR on LJ; however, one strain harboring I491>F mutation was susceptible to MGIT DST (Table 1). All the H445>L strains (*n* = 9/9) were resistant on LJ DST; however, only one (*n* = 1/9) was resistant on MGIT DST, while H445C was resistant on both LJ (5/6) and MGIT (4/6) DSTs in most of the strains. D435Y and L452P LJ DST reported most strains as resistant (16/21) and (15/20), respectively, while MGIT reported only (4/21) and (3/20) strains, respectively. Strains harboring L430P and H445N LJ DST showed some RR (6/20) and other sensitivity (7/18). In contrast, MGIT DST reported a higher discordance in RR (1/20) and sensitivity (3/18), respectively. In our strains, L430R was identified in two strains, and both were susceptible to LJ and MGIT DST. However, strains harboring double mutations L430R+D435Y are resistant on LJ (*n* = 3, 29.24%–100%), but MGIT shows two resistant 9.43%–99.16% and one sensitive. The resistance is due to D435Y mutations, not L430R (Table S1). The strains harboring H445N+S450W double mutations (*n* = 2) also exhibited discordance on MGIT (*R* = 1, *S* = 1), while LJ reported both as resistant (Table S1).

There were 30 mutations, including 20 combinations of mutations, 1 insertion (Ins), 3 deletions (Del), and 6 substitutions detected in *rpoB*, demonstrating concordant and discordant results on LJ and MGIT DST (Table S1). Among the 20 combinations of mutations, eight showed discordant RR resistance on LJ and MGIT (Table S1). Five samples harboring M434V+H445N mutations were RR on LJ (sensitivity 95% CI 47.82%–100.00%) and showed discordance in which one was sensitive on MGIT DST (sensitivity 95% CI 38.36%–99.49%). Similarly, one RR isolate harboring a double mutation (M434L+H445N) was detected RR on LJ (sensitivity 95% CI: 2.50%–100.00%) and demonstrated discordance on MGIT (sensitivity 95% CI: 0.00%–97.50%). DST was repeated for this double mutation, and the discordance results remained unchanged.

Ten isolates containing different combinations of mutations (two to three per combination), including two long resistant combinations T427I+D435Y+I491L and S428G+H445N+L452P, showed concordant results on both LJ and MGIT DST. Overall, four mutations, L430R (*n* = 2), N438K (*n* = 1), T444A (*n* = 1), and S431G (*n* = 1), were sensitive on both types of phenotypic DST in which T444A is novel in the current study. One novel large deletion, ggaccagaacaacccg/G, caused Del of amino acids (aa) DQNNP (435–439) showing concordant results of resistance on LJ and MGIT DST. One single amino acid, Del (Del of N at 519), and insertion (R at 432) were also resistant on both phenotypic DST. A single isolate harboring double mutations (H445>P+K446>Q) was resistant on both phenotypic DST (Table S1). However, to our knowledge, this double mutation in a single isolate is novel in our study. Mutations at Q436H+N437del (Table S1) and S428I were sensitive on MGIT DST at a critical concentration, while both of these were resistant on LJ DST and were classified as disputed mutations on MGIT DST. These mutations are also novel (Q436H+N437del, S428I) and were not previously reported as discordant. Double mutations S428T+D435G and S428R+H445Y, which show concordant results on both types of DST, are also a novel combination in a single RIF-resistant isolate for which the minimum inhibitory concentration (MIC) has not been determined.

MIC of the mutant *rpoB* was determined on MGIT and LJ (Table 2). Sixteen isolates harboring different discordant mutations exhibited MIC greater than 1 µg/mL; 12 had less than 1 µg/mL (<1 µg/mL) on MGIT, whereas 25 mutant RpoB isolates showed MIC greater than 40 µL/mL and 4 less than 40 µL/mL on LJ. The MIC of some samples harboring mutation His445Asn and Leu452Pro is still under processing on MGIT and LJ. Some of these have been sent to whole-genome sequencing (data are not available) to find some compensatory mutations. Asp435Tyr is a disputed mutation resulting in discordance results on liquid and solid DSTs. In order to look closely, we performed MIC on LJ and MGIT for four strains and WGS for two strains (BioSample Accession SAMN34126583). One strain with MIC > 1 on MGIT DST and >40 on LJ medium was closely analyzed on WGS and found another mutation Pro358Leu in the rpoB region, which resulted in higher MIC. Similarly for Leu430Pro, a second mutation was found Cys701Trp (BioSample accession SAMN34126582) which resulted in increased MIC > 1 on MGIT and >40 on LJ medium. For a more strongly disputed mutation Leu452Pro, a second mutation was detected at His445Tyr (BioSample accession SAMN34126584) causing increased MIC > 1 on MGIT and >40 on LJ medium.

**TABLE 2:** RpoB mutations and MICs of RIF

Amino acid change	Total	MIC > 1 (MGIT)	MIC = 0.5 (MGIT)	MIC = 0.25 (MGIT)	MIC = 0.12 (MGIT)	MIC = 0.06 (MGIT)	INV/CONT.	MIC >40 (LJ)	MIC = 20 (LJ)	MIC = 10 (LJ)	MIC = 05 (LJ)	INV/CONT.	WGS^*a*^
Asp435Tyr	4	1	1	1	1	0	0	2	0	1	1		2
Asp435Val	4	4	0	0	0	0	0	4	0	0	0		
His445Asn	4	0	2	0	0	0	2	1	1	1	0	1	1
His445Asp	2	2	0	0	0	0	0	2	0	0	0		
His445Cys	1	0	1	0	0	0	0	1	0	0	0		
His445Leu	2	2	0	0	0	0	0	2	0	0	0		
His445Tyr	2	2	0	0	0	0	0	2	0	0	0		
Ile491Phe	1	1	0	0	0	0	0	1	0	0	0		
Leu430Pro	2	1	1	0	0	0	0	2	0	0	0		1
Leu452Pro	4	1	1	1	1	0	0	2	0	0	0	2	2
Ser450Leu	4	4	0	0	0	0	0	4	0	0	0		1
Ser450Trp	2	0	0	0	0	0	0	2	0	0	0		
Total	32	16	4	4	2	2	2	25	1	2	1	3	7

^*a*^ WGS, whole genome sequencing.

## DISCUSSION

The prevalence of discordant (generally missed by rapid phenotypic DST methods) *rpoB* mutations is largely limited, and only a few studies addressed this problem and its impact on clinical implications (15, 31, 32). Population-based molecular studies of RIF mutation screening are required for better understanding and treatment options, particularly in early MDR.

In our study, the discordance rate of L430>P on MGIT and LJ seems high, while the previous study reported it “not associated” with MGIT DST and minimal confidence grading on solid DST (32). Some other previous studies have reported this mutation as discordant (9, 15, 19, 33 – 36). The frequency of *rpoB* mutations, including L430>P, may vary from geographic to treatment histories, as evidenced by Hong Kong and Bangladesh (37, 38). In Hong Kong, L430>P (*E. coli:* L511P), L452>P (*E. coli*: L533>P), and H445>L (*E. coli:* H526>L) represented 22% of all the mutations as compared to phenotypically RIF-resistant strains (<10% among all). However, the frequency of these three mutations was 18% (40/221) in Bangladesh retreatment cases. In the current study, the discordance of MGIT sensitivity of mutation L452>P (17/20, 85%) was much higher as compared to that of LJ (5/20, 25%). Miotto et al. reported MGIT sensitivity and moderate confidence grading on solid DST (32). Rigouts et al. reported these mutations in 14 strains that MGIT DST missed entirely; however, they were resistant to LJ (9). Similarly, other studies have reported this mutation as discordant (9, 15, 19, 33 – 35, 39).

Similarly, the D435>Y sensitivity frequencies were also different on MGIT and LJ. The confidence grading of this mutation is unavailable on MGIT DST, while a moderate confidence grading has been given on solid DST (32). Rigouts et al. detected D435>Y in six resistance strains, completely missed on MGIT DST (9); however, they were resistant on LJ DST. The possible reason for these variations may be due to compensatory mutations (40) responsible for the fitness cost in different media. Similarly, other studies have reported this mutation as discordant (9, 15, 33 – 36).

A more recent study reported RIF resistance from more than 31k genome sequence analyses, in which Ser450>Leu (15.2%), Asp435>Val (1.8%), and His445>Tyr (1.3%) (40) were the most common RpoB mutations. In the current study, 18 isolates harbor mutation H445>N, of which 15 (83.3%) were MGIT-sensitive and 11 (61.1%) were LJ-sensitive. The confidence grading for H445>N is not available on MGIT DST, while minimal confidence grading on solid DST was given in a previous study (32). Similar to our results, H445>N was also reported as a discordant mutation (9, 35, 39, 41). A more recent study (42) reported that H445>L, D435>Y, and L452>P are involved in a low level of RIF resistance (MIC 0.5–1.0 µg/mL), whereas H445>N and L430>P lower MICs 0.25–0.5 µg/mL under the epidemiological cutoff (MIC 0.125 µg/mL). H445>L is also discordant (9, 15, 35, 36, 41), and only one out of nine (11.1%) was MGIT-resistant; however, all nine isolates were LJ-resistant. H445>L mutants demonstrated a high confidence grading on solid DST (9, 32). Liu et al. (43) also detected these (H445>L, D435>Y, and L452>P) in 173 MDR-TB with various frequencies, i.e., 4%, 1%, and 3.5%, respectively, in China. This difference in high discordance results in MGIT DST may lead to misdiagnosis and mistreatment of MDR cases.

Siu et al. reported that 4% of RIF resistance cases do not harbor mutations in RRDR. In the current study, two mutations (I491>F and V170>F) were detected outside the RRDR of RpoB in which I491>F was detected in five RIF resistance isolates, and only one (20%) was MGIT-sensitive; however, all five isolates were LJ-resistant, which have been classified in minimal confidence grading (35, 44). The mutant V170>F showed concordance results on LJ and MGIT. According to a WHO multi-country surveillance study, V170>F and I491>F have elevated RIF MICs in *M. tuberculosis* [0.5% (95% CI 0.2%**–**1.2%)] (45 – 47). However, I491>F (*E. coli* I572F) have been accounted for more than half of RR in Eswatini, while its frequency in South Africa, a neighboring country, was <1% (48, 49). Several studies reported the MIC for mutants I491F and V170>F as 100 and 125 µg/mL, respectively (50 – 52). One notable point from Ma et al.’s study is the experimental proof of compensatory effects of non-RRDR mutations, which may have molecular markers’ importance in predicting the fitness of clinical *M. tuberculosis* strains.

*M. tuberculosis* isolates harboring double and triple mutations have also shown discordant results on MGIT DST only; however, all these strains were resistant on LJ DST (Table S1). A single triple mutation isolate (S428G+H445N+L452P) exhibited concordance results on both phenotypic DSTs. This combination is also novel and has not been reported in earlier studies. Mutation D435G, detected in one RIF-resistant strain, is associated with high-level resistance on LJ but not MGIT (32). However, we reported this mutation as discordance on LJ and MGIT. Double mutations M434V+H445N (*n* = 5) and L430R+D435Y (*n* = 2) were all LJ-resistant. Double mutants (M434V+H445N and L430R+D435Y), which showed different frequencies on MGIT and LJ resistance, were reported as MGIT-resistant only without performing LJ DST (32). Miotto et al. (32) reported M434I+H445N mutant as undisputed and MGIT-resistant without confirmation on LJ. However, isolates harboring double mutation (M434I+H445N) are RR on both LJ and MGIT DSTs; even DST was repeated twice for these isolates. Previously, L430R was reported as a low-level resistance mutation to RIF, and S431G is sensitive and has no effect on RIF interaction with RpoB (53). In an earlier study (43), L430>R and L430>P were detected in MDR isolates and other RpoB mutations at positions 431, 435, and 445, except for four isolates where L430P was detected alone. Mutation N438K (*n* = 1), which was found LJ- and MGIT-sensitive, was not classified (unpublished) in WHO technical report 2021 (44). One novel mutation (T444A), LJ- and MGIT-sensitive, is also missing in WHO report 2021 on the critical concentration of drugs.

The frequencies of double discordance mutations are low, and one could comment that the data are insignificant; however, in either case, MDR-TB misclassification could not be ignored to achieve the Global TB Control Program. Let us consider that such instances are very rare. Still, if a TB outbreak occurs because of strains harboring these resistance mechanisms, it will cause misclassification, leading to mistreatment and further transmission in populations.

Only H445>P and a triple mutation in a single isolate T444>T+H445>P+K446>Q have been reported in a previous study, associated with MGIT resistance (32). However, to our knowledge, this double mutation (H445>P+K446>Q) in a single isolate is novel in our study, conferring resistance on both LJ and MGIT. Mutations 450>L, 435>V, 450>W, 445>D, and 445>Y are associated with a high level of RIF resistance in previous studies (9, 15, 33 – 36). Mutations F433ins, 432K, 432P, 432L, and H445R were also given high confidence grading on liquid and solid DSTs (54). However, H445>G (*n* = 1) is a globally rare mutant that exhibited discordance DST. Previous studies also reported it as discordant and low frequency, associated with RR (55 – 57).

Our results show that both LJ and MGIT DSTs are prone to miss rifampicin resistance; however, the discordance rate in MGIT DST is higher than that in LJ. Our study population represents a substantial number of common and less common mutations and their sensitivity on LJ and MGIT DSTs. Rigouts et al. (9) showed that the automated MGIT DST is more prone to demonstrate rifampicin-susceptible results in discordant *rpoB* mutations than LJ DST. Similarly, a study involving the Supra-National TB Reference Laboratories network of the WHO has shown that more RIF-susceptible results were observed in liquid DST compared to solid DST in samples with discordance *rpoB* mutations (15). Miotto et al. hypothesized that discordant mutations lower the RIF binding affinity but do not prevent it completely (32). Some other studies pointed out that a loss of fitness in *M. tuberculosis* strains may slow their growth (58, 59), while MGIT is a fast-growth system. The L430>P, D435>Y, H445>C/L/N, and L452>P mutations have already been reported as disputed mutations on MGIT (9, 32, 43, 53), and isolates harboring it will be misclassified on phenotypic results (MGIT).

About 14% of all *rpoB* mutations are discordant (17) when subjected to conventional phenotype-based drug susceptibility testing. The *M. tuberculosis* isolates with discordant mutations are thus classified as RIF-susceptible, and the patients are treated as drug-susceptible TB (16, 60), leading to poor treatment outcomes (16, 17, 39). Many researchers suggested that the critical concentration for MGIT DST should be lowered as low as 0.0625–0.5 µg/mL (39, 44, 61). Though earlier studies showed that performing MGIT DST at a critical concentration of 0.25 µg/mL failed to address the problem as some discordant isolates were still missed, some susceptible isolates were classified as resistant (36).

Recently, the WHO has revised rifampicin critical concentration from 1 µg/mL to 0.5 µg/mL on MGIT DST to reduce this issue (44). The misclassification rate of RIF resistance isolates harboring discordant mutations may be higher for MGIT DST than for LJ due to the new MGIT critical concentrations, which need further clarification. There will be a higher overlap between the MIC of discordant resistant and susceptible isolates with MGIT as it has a shorter incubation period (33).

WHO found six RRDR mutations L430>P, D435>Y, H445>L, H445>N, H445>S, and L452>P and some outside the RRDR (non-RRDR). We identified some mutations outside RRDR (170>F, 491>L, and 491>F), which cannot be disregarded as GeneXpert testing services are decentralized, and RR cases are only systematically referred for DST. Therefore, samples from patients reported as rifampicin-sensitive on GeneXpert but with poor response to treatment should be referred for LJ DST or gene sequencing to avoid misdiagnosis of resistant strains; otherwise, ineffective treatment will result in proliferation and transmission of *M. tuberculosis* strains in the population. Mutations with significant frequencies outside the RRDR region may be considered in genotypic DST, as evidenced by a previous study in which 30% of RR cases were detected outside the RRDR (62) region.

The variability in MICs with the same mutation (Table 2) may be due to the growth rate (63). The growth rate has been associated with mutations, a useful indicator of fitness costs (64). *M. tuberculosis* isolates with a known RR mutation have comparable fitness costs irrespective of the genetic makeup. Many factors impact *M. tuberculosis* fitness costs, including mutations in *rpoA* and *rpoC,* which compensate for RIF resistance behind mutations in RpoB (65). Thus, the *in vitro M. tuberculosis* growth rate is significantly improved in the presence of *rpoB* mutations along with compensatory mutations. Moreover, *M. tuberculosis* isolates harbor mutations in other targets conferring resistance, which may sustain uncertain fitness costs. Estimating accurate fitness costs is difficult as the diversity of mutations under different environmental conditions may also have diverse impacts on fitness. Therefore, precision in determining the MIC with variable growth rates may not be possible. Moreover, some studies closely look only for a single mutation, so double mutations or mixed population is overlooked. In the case of well-known disputed mutations (430>Pro, 435>Tyr, 441>Leu, 445>Asn, 445>Leu, 452>Pro, and 491>Phe), if found resistant on MGIT DST, a close look using deep sequencing should be applied to explore any other mutations that may cause increased MIC.

### Recommendation

It is also important to note that some mutations are geographically specific, which may not be in MGIT detection but have a significant frequency in *M. tuberculosis* isolates. This has been evidenced by our recent study (66) from the same geography of Khyber Pakhtunkhwa Province, in which we reported the majority of MDR-TB isolates to have a high frequency of *rpoC* mutations (32/46), a locus recognized to restore the fitness of RIF resistance. Purely genotypic DST with untargeted whole-genome sequence for MGIT susceptibility and LJ resistance, as recently initiated by the United Kingdom (67), may be useful for countries with high-burden TB. Alternatively, rpoB, rpoC, and rpoA sequencing of MGIT-sensitive and LJ-resistant isolates might be useful for reducing the gap in RIF resistance and for GeneXpert comparison. Moreover, geographic-specific mutations may play a role in evading the detection power of current MGIT, as discussed in the text. Identifying the complete range of RIF resistance and compensatory mutations will improve clinical management and surveillance decision-making using long-read sequencing data. While genotypic DST with untargeted WGS will be an important initiative, long-read WGS (lrWGS) (68) is most important to capture the compensatory mutations. In a programmatic setting, we would support the implementation of lrWGS for MGIT and targeted genotypic wild-type isolates to reduce and unveil the discordance and compensatory mutations associated with drug resistance. Such steps may be useful to reduce the TB and drug resistance burden.

### Conclusion

Numerous discordant and concordant novel mutations and their combination have been detected on LJ and MGIT DST. Mutations N438K and T444A, sensitive on LJ and MGIT DSTs, are novel. Two novel discordant combinations, Q436H+N437del and S428I, were also detected as LJ-resistant and MGIT-sensitive. One novel large deletion (Del) ggaccagaacaacccg/G (Del of DQNNP at positions 435–439) demonstrated concordance on LJ and MGIT DST. Numerous double and triple mutations, including some novel combinations, have also shown higher discordant results on MGIT DST. The discordant level in MGIT DST is more than that in LJ DST, which may cause poor treatment outcomes. In discordant mutations, phenotypic DST should be replaced and preferred with RIF target genes or untargeted whole sequencing. All clinicians involved in TB treatment management should be well trained to tackle such discordance mutations for proper treatment regimens. The gold standard for rifampicin resistance detection may be reconsidered as a known link between discordant mutations and poor clinical outcomes. Isolates showing discordance on MGIT may undergo sequencing to unveil the hidden resistance mechanism.

## Data Availability

Whole-genome sequencing (WGS) data are available under BioSample (accession SAMN34126582, SAMN34126583, and SAMN34126584). Sanger sequencing results are available at NCBI GenBank: BankIt2723841: OR290177–OR290272; BankIt2723888: OR290273–OR290370; BankIt2723951: OR290371–OR290468; BankIt2723992: OR290469–OR290566; BankIt2723996: OR290567–OR290597.

## AFTER EPUB

*[This article was published on 20 November 2023 with an error in the author affiliations. The error was corrected in the current version, posted on 14 December 2023.]*

## SUPPLEMENTAL MATERIAL

Table S1 spectrum.01631-23-s0001.docx Summary of rifampicin concordance and discordance on LJ and MGIT DST, sensitivity, stratified by *rpoB* mutation type for *M. tuberculosis* isolates.

ASM does not own the copyrights to Supplemental Material that may be linked to, or accessed through, an article. The authors have granted ASM a non-exclusive, world-wide license to publish the Supplemental Material files. Please contact the corresponding author directly for reuse.
