# Supplementary material for: Phenotype versus genotype discordant rifampicin susceptibility testing in tuberculosis: implications for a diagnostic accuracy
Source: Microbiol Spectr. 2023 Nov 20;12(1):e01631-23. doi: 10.1128/spectrum.01631-23 (PMC10783056; doi:10.1128/spectrum.01631-23)
Supplement: Table S1 — Summary of rifampicin concordance and discordance on LJ and MGIT DST, sensitivity, stratified by rpoB mutation type for M. tuberculosis isolates. [file spectrum.01631-23-s0001.docx]

Table S1: Summary of rifampicin concordance and discordance on LJ and MGIT DST, Sensitivity, stratified by *rpoB* mutation type for *M. tuberculosis* isolates

|  | **Codon Change, *M. tuberculosis* numbering (*E. Coli* numbering)** |  | **Rifampicin results on LJ DST** | | | **Rifampicin results on MGIT DST** | | | **Total** |
| --- | --- | --- | --- | --- | --- | --- | --- | --- | --- |
| **S.No** |  | **Amino Acid Change**  ***M. tuberculosis* numbering (*E. Coli* numbering)** | **R**  **(%)** | **S**  **(%)** | **Sensitivity 95% CI** | **R(%)** | **S(%)** | **Sensitivity 95% CI** |  |
|  | atg→gtg, cac→aac | M434V + H445N  (M515V + H526N) | 5  (100) | 0(0) | 47.82 to 100.0 | 4(80) | 1(20) | 28.36 to 99.49 | 5 |
|  | ctg→cgg, gac→tac, | L430R + D435Y  (L511R + D516Y) | 3  (100) | 0(0) | 29.24 to 100 | 2  (66.6) | 1  (33.3) | 9.43 to 99.16 | 3 |
|  | atg→ata, cac→aac | M434I + H445N  (M515I + H526N) | 2  (100) | 0(0) | 15.81 to 100 | 2(100) | 0(0) | 15.81 to 100.00 | 2 |
|  | cac→**aac,** tcg→tgg | H445N + S450W  (H526N + S531W) | 2  (100) | 0(0) | 15.81 to 100.00 | 1(50) | 1(50) | 1.26 to 98.74 | 2 |
|  | **g**gaccagaacaacccg/G | Del DQNNP from 435-439 (inframe) (Del of DQNNP from 516-520 (inframe) | 1  (100) | 0(0) | 2.50 to 100.00 | 1(100) | 0(0) | 2.50 to 100.00 | 1 |
|  | **c**accagccagctg/**C** | Del of aa TSQL from 427-30 (inframe)  (del of TSQL from 508-511 (inframe) | 1  (100) | 0(0) | 2.50 to 100.00 | 1(100) | 0(0) | 2.50 to 100.00 | 1 |
|  | **a**aac/A at 1312-1314 | del of N at 438  (Del of N at 519) | 1  (100) | 0(0) | 2.50 to 100.00 | 1(100) | 0(0) | 2.50 to 100.00 | 1 |
|  | acc→**acg, cac**→ccc,  aag→cag | H445P + K446Q  (H526P + K527Q) | 1  (100) | 0(0) | 2.50 to 100.00 | 1(100) | 0(0) | 2.50 to 100.00 | 1 |
|  | acc→atc, gac→tac, atc→ctc | T427I + D435Y + I491L (T508I + D516Y + I572L) | 1  (100) | 0(0) | 2.50 to 100.00 | 1(100) | 0(0) | 2.50 to 100.00 | 1 |
|  | Agaa/A at 1308-10 | Q436H + N437del  (QN517H) | 1  (100) | 0(0) | 2.50 to 100.00 | 0(0) | 1(100) | 0.00 to 97.50 | 1 |
|  | agc→acc, gac→ggc | S428T + D435G  (S509T + D516G) | 1  (100) | 0(0) | 2.50 to 100.00 | 1(100) | 0(0) | 2.50 to 100.00 | 1 |
|  | agc→agg, cac→tac | S428R + H445Y  (S509R + H526Y) | 1  (100) | 0(0) | 2.50 to 100.00 | 1(100) | 0(0) | 2.50 to 100.00 | 1 |
|  | agc→agg, ctg→ccg | S428R + L430P  (S509R + L511P) | 1  (100) | 0(0) | 2.50 to 100.00 | 0(0) | 1(100) | 0.00 to 97.50 | 1 |
|  | agc→ggc, cac→aac,  ctg→ccg | S428G + H445N + L452P (S509G + H526N + L533P) | 1  (100) | 0(0) | 2.50 to 100.00 | 1(100) | 0(0) | 2.50 to 100.00 | 1 |
|  | atg→ata, gac→ggc, gcg→gtg, | M434I + D435G + A451V (M515I + D516G + A532V) | 1  (100) | 0(0) | 2.50 to 100.00 | 0(0) | 1(100) | 0.00 to 97.50 | 1 |
|  | atg→ctg, cac→aac | M434L + H445N  (M515L + H526N) | 1  (100) | 0(0) | 2.50 to 100.00 | 0(0) | 1(100) | 0.00 to 97.50 | 1 |
|  | caa→aaa, cac→gac | Q432K + H445D  ( Q513K + H526D) | 1  (100) | 0(0) | 2.50 to 100.00 | 1(100) | 0(0) | 2.50 to 100.00 | 1 |
|  | cac→aac, ctg→cag | H445N + L452Q  ( H526N + L533Q) | 1  (100) | 0(0) | 2.50 to 100.00 | 1(100) | 0(0) | 2.50 to 100.00 | 1 |
|  | cag→cac , cac→cgc | Q429H + H445R  (Q510H + H526R) | 1  (100) | 0(0) | 2.50 to 100.00 | 1(100) | 0(0) | 2.50 to 100.00 | 1 |
|  | ctg→ccg , cac→aac | L430P + H445N  ( L511P + H526N) | 1  (100) | 0(0) | 2.50 to 100.00 | 1(100) | 0(0) | 2.50 to 100.00 | 1 |
|  | gac→ggc , gcg→gtg | D435G + Ala451V  ( D516G + A532V) | 1  (100) | 0(0) | 2.50 to 100.00 | 0(0) | 1(100) | 0.00 to 97.50 | 1 |
|  | gac→tac , aac→cac | D435Y + NN437H  ( D516Y + N518H) | 1  (100) | 0(0) | 2.50 to 100.00 | 1(100) | 0(0) | 2.50 to 100.00 | 1 |
|  | Ins cgc 1294, | Ins of R at 432 ( R513 Ins (inframe)) | 1  (100) | 0(0) | 2.50 to 100.00 | 1(100) | 0(0) | 2.50 to 100.00 | 1 |
|  | WT |  | 0(0) | 120  (100) |  | 0(0) | 120  (100) |  | 120 |

R= Resistant, S= Susceptible, CI= Confidence Interval, Ind = Indeterminate
